# The development of PARP inhibitors in ovarian cancer: from bench to bedside

**DOI:** 10.1038/bjc.2015.394

**Published:** 2015-12-15

**Authors:** Yvette Drew

**Affiliations:** 1 Northern Institute for Cancer Research, Newcastle University, Newcastle upon Tyne, UK; 2 Northern Centre for Cancer Care, Freeman Hospital, Newcastle upon Tyne, UK

**Keywords:** olaparib, PARP, ovarian cancer

## Abstract

The nuclear enzyme poly (ADP-ribose) polymerase (PARP) represents an important novel target in the treatment of ovarian cancer. This article charts over 50 years of research from the discovery of the first PARP enzyme in 1963, to the approval and licensing in 2015 of the first PARP inhibitor, olaparib (Lynparza), in the treatment of *BRCA-*mutated ovarian cancer.

Ovarian cancer is the fifth most common cancer in women in developed countries, accounting for 140 000 deaths per year worldwide (World Health Organization, 2008; Siegel *et al*, 2012). The majority of women present with advanced-stage (3 or 4) disease, where 5-year survival rates are poor at around 27% (Siegel *et al*, 2012). Despite initial high responses to platinum-based chemotherapy and cytoreductive surgery, more than 70% of these patients will relapse with limited subsequent treatment options (Hanker *et al*, 2012). There is a pressing need for improved treatments that can extend survival, delay disease progression and maintain quality of life for patients with ovarian cancer.

A better understanding of cancer is leading to the identification of distinct cancer molecular sub-types, new anticancer targets, and more individualised patient treatment approaches. The development of poly(ADP-ribose) polymerase (PARP) inhibitors for the treatment of *BRCA*-mutated (*BRCA*m) ovarian cancer is an example of this approach in action. This review summarises the research behind this development; charting the discovery of the first PARP enzyme (Chambon *et al*, 1963) and the development of PARP inhibitors as a class; highlighting why cancers defective in DNA repair could be selectively sensitive to these agents, and why the approval of the PARP inhibitor olaparib (Lynparza) has changed the management of *BRCA*m ovarian cancer.

## DNA damage response, repair pathways and *BRCA*

The accurate and efficient repair of DNA damage is essential for cells to function and maintain genomic stability (Hoeijmakers, 2001). In humans, acquired or inherited defects in DNA damage response and repair pathways can result in an increased lifetime risk of cancer (Hoeijmakers, 2009). DNA double-strand breaks (DSBs) are regarded as the most lethal of the DNA insults and, if left unrepaired, result in genomic instability, carcinogenesis and ultimately cell death (Hoeijmakers, 2001). DNA DSBs can arise as a result of direct damage to both strands of DNA from exogenous agents, such as ionising radiation or chemotherapy (Helleday *et al*, 2008), or as part of normal cell physiology, for example, to permit genetic recombination during meiosis (Neale and Keeney, 2006) and the rearrangements needed for the development of immunoglobulin genes during V(D)J (variable, diversity and joining) recombination (Leavy, 2010).

**Figure 1:**
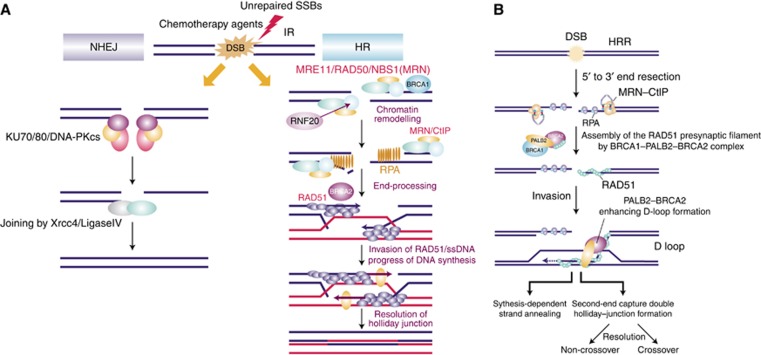
**Mechanisms of DNA double-strand break (DSB) repair.** Double-stranded breaks in DNA are typically repaired through one of two pathways: (**A**) non-homologous end joining (NHEJ); (**A, B**) homologous recombination (HR). Proteins involved in NHEJ include KU70/80, DNA-PKcs, XRCC4 and DNA ligase IV. Proteins involved in HR include MRE11, RAD50 and NBS1 (which form the MRN complex); CtIP; RNF20; RPA; RAD51; PALB2; BRCA1 and BRCA2. Abbreviations: HRR, homologous recombination repair; IR, ionising radiation; SSB, single-strand break; ssDNA, single-stranded DNA. Note: (**A**) Reproduced with permission from Pioneer Bioscience Publishing Company (© Saito *et al*, 2013). (**B**) Reprinted with permission from Nature America, Inc. (© Buisson *et al*, 2010).

The two primary DSB repair pathways in humans are non-homologous end joining (NHEJ) and homologous recombination repair (HRR). These two pathways operate independently but do share some common proteins (Figure 1). The pathway that is used to repair the DNA damage depends principally on the origin of the DSB and the stage in the cell cycle in which the DSB occurs (Takata *et al*, 1998). The preferred pathway is HRR, as it is an error-free pathway; however, it is dependent on the availability of sister chromatids and can only take place in late S and the G_2_ phases of the cell cycle (O'Driscoll and Jeggo, 2006). A significant number of DSBs can also arise during DNA replication when a replication fork encounters an unrepaired, single-strand break (SSB); the HRR pathway and the nuclear enzyme PARP-1 have a vital role in repairing these DSBs (Bryant *et al*, 2009; Helleday *et al*, 2007). Homologous recombination repair involves a variety of proteins, including BRCA1 and BRCA2. BRCA1 has a role in signalling of the DNA DSB damage response and subsequent repair via HRR, but also in transcriptional regulation and cell-cycle checkpoint control; whereas BRCA2 has a more direct repair role in HRR through its regulation of Rad51 (Gudmundsdottir and Ashworth, 2006). It is proposed that the BRCA2–Rad51 complex binds to the exposed DNA, and this binding then enables the loading of Rad51 onto the break and the formation of the presynaptic filament (Yang *et al*, 2002). Given the functions of BRCA1 and 2, it would be logical to hypothesise that deficiencies within either gene will result in defective HRR and subsequent loss of efficient and effective DNA DSB repair.

## *BRCA* mutations and ovarian cancer

The *BRCA1* gene was identified in 1990 by Mary King’s group working at Berkeley, CA, USA. The name BRCA was originally chosen to stand for Berkeley California, but was later changed to represent breast cancer susceptibility (Hall *et al*, 1990). The gene was subsequently cloned in 1994 by Myriad Genetics (Miki *et al*, 1994). Around the same time, the *BRCA2* gene was discovered by Stratton and Wooster working at the Institute of Cancer Research, London, UK (Wooster *et al*, 1994). The identification of these genes represented a significant breakthrough in the management of breast and ovarian cancer families, enabling the introduction of risk assessment, genetic counselling and *BRCA* mutational analysis. Subsequently, over 2000 distinct mutations and sequence variations in the *BRCA* genes have been identified (Audeh, 2014), with *BRCA1* mutations more common, occurring approximately twice as frequently as *BRCA2* (Chen and Parmigiani, 2007).

Women who inherit a deleterious *BRCA1* or *BRCA2* mutation have up to a 40% and 20% lifetime risk, respectively, of developing ovarian cancer, and higher risks of developing breast cancer (Chen and Parmigiani, 2007). The prevalence of germline (g) *BRCA* mutations in ovarian cancer has historically been estimated to be around 10–15% (Risch *et al*, 2001). However, recent reports suggest that this may be a gross underestimate, especially in women with high-grade serous ovarian cancer (HGSOC) (Risch *et al*, 2006; Cancer Genome Atlas Research Network, 2011; Alsop *et al*, 2012). In addition, in one series where 17% of patients with HGSOC were found to carry a *BRCA* mutation, almost half (44%) of these women had no family history of cancer (Alsop *et al*, 2012). Such data support the use of *BRCA* mutation testing in all patients with HGSOC, regardless of family history. This expansion in *BRCA* testing will require changes to the traditional genetic service pathways in which patients are screened and referred based on family history, moving to a more streamlined oncology-based genetic testing service.

Over the past two decades the main focus in the treatment of women identified as *BRCA* mutation carriers has been ovarian and breast cancer prevention through prophylactic surgery, and early cancer detection through screening (Domchek *et al*, 2006). However, surveillance and surgery will not prevent all carriers developing cancer and many already have cancer at the time their mutation status is diagnosed. The current management of *BRCA*m-associated ovarian cancer is not different to the treatment of the non-*BRCA* stage-matched cases. However, recent data suggest that these *BRCA*m cancers should be treated as a distinct disease entity and that *BRCA* mutation status has a major influence on ovarian cancer patient outcomes. *In vitro* studies have demonstrated that *BRCA1*- and *2*-deficient cells are more sensitive than their wild-type controls to platinum analogues and less sensitive to anti-microtubule agents, such as the taxanes (Bhattacharyya *et al*, 2000; Tassone *et al*, 2003; Tan *et al*, 2008). Data from 26 observational clinical studies of 3879 women with ovarian cancer reported that those with *BRCA*m cancers have a better outcome following cytoreductive surgery and platinum-based chemotherapy than their non-*BRCA*m counterparts, with prolonged progression-free and greater 5-year overall survival (Bolton *et al*, 2012). A recent meta-analysis of 14 ovarian cancer studies has confirmed this, showing that *BRCA* status in ovarian cancer is an independent predictor of outcome (Zhong *et al*, 2015). In the relapsed setting, *BRCA*m carriers have also been shown to respond better to both platinum- and non-platinum-containing regimens (Alsop *et al*, 2012).

Knowing the *BRCA* mutation status of a patient with ovarian cancer is important in terms of managing individual risk and identifying other family members at risk. In addition, a patient’s *BRCA1* and *2* mutation status can now inform the physician and patient regarding treatment outcomes, and, with the development of PARP inhibitors, offers patients the potential for personalised anticancer treatment.

## Poly (adp-ribose) polymerase and the development of PARP inhibitors

**Figure 2:**
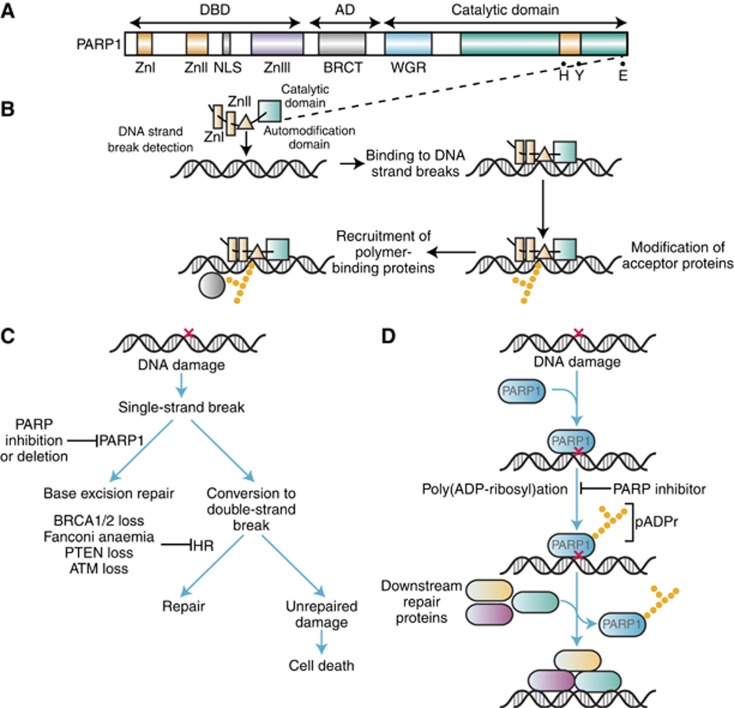
**Base-excision repair/single-strand break pathway.** (**A**) Structure of PARP1. (**B**) Activation of PARP1 in response to DNA damage. After binding to damaged DNA, the enzymatic activity of PARP1 increases following a conformational change to the active site. PARP1 synthesises poly(ADP) ribose chains that alter protein function and recruit additional proteins. (**C**) Role of PARP1 in base excision repair. (**D**) Model showing recruitment of DNA repair proteins following DNA damage. Abbreviations: AD, automodification domain; BRCT, *BRCA1* C-terminal domain; DBD, DNA-binding domain; HR, homologous recombination; NLS, nuclear localisation signal; WGR, tyrptophan–glycine–arginine-rich domain; Zn, zinc finger. Note: Reproduced with permission from the American Society of Clinical Oncology (© Scott *et al*, 2015).

The discovery of the first PARP was made over 50 years ago when researchers in Paul Mandel’s laboratory observed the synthesis of a new polyadenylic acid after adding nicotinamide mononucleotide to rat liver extracts (Chambon *et al*, 1963). By 1980 it was known that this nuclear enzyme, PARP-1, was activated by DNA damage and played a pivotal role in the repair of DNA SSBs via the base-excision repair/single-strand break repair (BER/SSBR) pathway (Figure 2) (Benjamin and Gill, 1980). Seminal work by Sydney Shall’s group subsequently demonstrated that PARP-1 was not only involved in the repair of SSBs, but inhibiting it could enhance the cytotoxic effects of methylating agents in leukaemic mice cells (Durkacz *et al*, 1980), suggesting that PARP inhibitors could act as chemosensitisers. There are now 17 members of the PARP nuclear superfamily and it is PARP-1 and 2 that are involved in DNA repair (Rouleau *et al*, 2010).

The first inhibitor of PARP, 3-aminobenzamide (3-AB), was identified over 30 years ago following the observation that nicotinamide and 5-methylnicotinamide competed with NAD+ as a PARP substrate (Purnell and Whish, 1980). Poly (ADP-ribose) polymerase inhibitor development pipelines initially investigated the potential for PARP inhibition to act as potentiators of chemotherapy and radiotherapy (Ferraris, 2010). More recently, they have pursued their therapeutic application as single agents, selectively killing cells with defects in DNA repair pathways, such as those with *BRCA1/2* mutations. There are currently four PARP inhibitors in Phase III development for ovarian cancer (Table 1). The most developed in the class is olaparib, a potent, oral inhibitor of PARP-1 and 2 that induces lethality in tumours with HRD, such as *BRCA1/2* mutations (Evers *et al*, 2008; Rottenberg *et al*, 2008). Olaparib is associated with significant clinical benefit in high-grade ovarian cancers with germline and/or somatic mutations within the *BRCA1/2* genes (Fong *et al*, 2009; Audeh *et al*, 2010; Tutt *et al*, 2010; Gelmon *et al*, 2011; Ledermann *et al*, 2014; Kaufman *et al*, 2015; Oza *et al*, 2015). This topic is reviewed within this Supplement (Ledermann, 2015). Why single-agent PARP inhibitors are active in *BRCA*m cancers is explained below through the concept of ‘synthetic lethality’.

## Poly (adp-ribose) polymerase inhibitors as single agents in *BRCA*m cancers—the concept of synthetic lethality

In 2005, two articles published in *Nature* reported that cells deficient in *BRCA1* and *2* were 100- to 1000-fold more sensitive to PARP inhibitors than *BRCA1/2* heterozygote or wild-type cell lines (Bryant *et al*, 2005; Farmer *et al*, 2005). Bryant *et al* used the PARP inhibitors NU1025 and AG14361, both forerunners to rucaparib (Clovis Oncology, Boulder, CO, USA). In mice xenografts, three out of five V-C8 tumours responded to a 5-day dosing of AG14361, with one mouse showing complete remission and no sign of tumour at autopsy. In addition, the articles reported an induction in *γ*H2AX foci formation (representing DNA DSBs) and Rad51 foci formation (indicating functional HR repair) in the XRCC1-deficient EM9 (Chinese hamster ovary) cell lines. In the V-C8 cells, an increase in *γ*H2AX foci formation, but not Rad51, was observed following exposure to NU1025.

In the *Nature* sister article, Farmer *et al* (2005) demonstrated the sensitivity of both *BRCA1-* and *BRCA2*-deficient cell lines to the specific inhibition of PARP-1 by two small-molecule inhibitors KU0058684 and KU0058948, forerunners to olaparib. They demonstrated that 24-h exposure to the PARP inhibitor resulted in permanent G2/M cell-cycle arrest or apoptosis. They also reported a three-fold increase in sensitivity over the DNA-damaging agent cisplatin for *BRCA1/2*-deficient cells. Both research groups independently concluded that *BRCA*-deficient cells were selectively sensitive to PARP inhibition by a mechanism of ‘synthetic lethality’.

**Figure 3:**
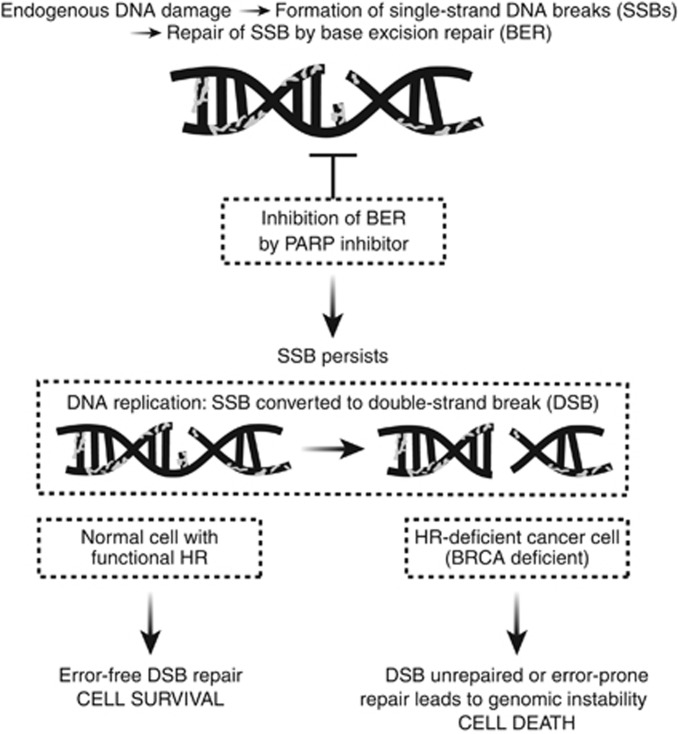
**Synthetic lethality of PARP inhibitors in *BRCA*-deficient cells.**

‘Synthetic lethality’ is the concept by which cancer cells are selectively sensitive to the inactivation of two genes or pathways when inactivation of either gene or pathway alone is non-lethal (Kaelin, 2005). This proposed mechanism of synthetic lethality of PARP inhibitors in *BRCA*-deficient cells is outlined in Figure 3. Poly (ADP-ribose) polymerase inhibition leads to the accumulation of DNA SSBs that result in unrepaired stalled replication forks and ultimately DSBs. These DNA DSBs are normally repaired by the HRR pathway (Hoeijmakers, 2001). In HRR-defective cells, that is, those with *BRCA1/2* mutations, these DSBs are left unrepaired or are repaired in an error-prone way by alternative non-homologous end-joining DNA repair; both outcomes can result in genomic instability and ultimately cell death. Whereas, in cells with functional HRR, that is, those with heterozygous mutations or wild-type *BRCA*, DSBs will be accurately and efficiently repaired, and inhibiting PARP will not result in cell death. Clinical trials are now confirming these preclinical data demonstrating that, as a class, PARP inhibitors are active in *BRCA*m cancers.

## Future directions for PARP inhibitors

The majority of ovarian cancers are not attributed to hereditary germline mutations in the *BRCA1* and *2* genes (Venkitaraman, 2002), so a key question is whether single-agent PARP inhibitors can be used to treat patients within the larger ovarian cancer population. It is known that HRD is not exclusive to germline *BRCA*m cancers, for example; molecular analysis of HGSOC as part of The Cancer Genome Atlas revealed that approximately 50% were shown to harbour HRD (Cancer Genome Atlas Research Network, 2011). This HRD included somatic *BRCA* mutations (6–8%) and epigenetic silencing in non-*BRCA* genes, such as *ATM* and *RAD51*. In addition, by using a functional assay of HRR, Mukhopadhyay *et al* (2010) demonstrated that 50% of primary cultures generated from ascites in unselected HGSOC patients had HRD and were sensitive to PARP inhibitors. Developing a diagnostic signature of HRD in cancers is the focus of the ongoing rucaparib studies (www.clinicaltrials.org). Preliminary results from the rucaparib ARIEL 2 study (NCT 01891344) indicate efficacy in patients who have *BRCA*m ovarian cancer, but also in those who are *BRCA* wild-type with high tumour genomic loss of heterozygosity (McNeish *et al*, 2014). The study hopes to develop a companion diagnostic to use within the ongoing Phase III trial (ARIEL 3; NCT01968213) of rucaparib in platinum-sensitive ovarian, fallopian tube or primary peritoneal high-grade cancer patients.

Another therapeutic approach is to induce HRD in otherwise HRR-competent cancers by altering the tumour microenvironment through hypoxia, or to combine PARP inhibitors with agents that can downregulate HRR, such as vascular endothelial growth factor (VEGF) inhibitors. This concept, known as ‘contextual’ synthetic lethality, could further broaden the application of this class of drugs and is the rationale behind many ongoing clinical trials. Preliminary data from a Phase II trial combining olaparib with the potent, oral VEGF tyrosine kinase inhibitor, cediranib, was shown to significantly improve progression-free survival over olaparib alone (9.0 months *vs* 17.7 months) (Liu *et al*, 2014); a confirmatory study is awaited.

Based on a wealth of preclinical data showing that PARP inhibitors potentiate the effects of DNA-damaging chemotherapy agents, such as the platinums, temozolomide and topoisomerase inhibitors (Delaney *et al*, 2000; Calabrese *et al*, 2004; Donawho *et al*, 2007), the original therapeutic intention of these agents was as chemopotentiators. Furthermore, inhibition of PARP has been shown to augment the antitumour activity of other agents that impair HRR, such as the DNA-synthesis inhibitor, gemcitabine (Virag and Szabo, 2002; Jacob *et al*, 2007). However, early clinical trials investigating multiple chemotherapy and PARP inhibitor combinations have reported enhanced myelosuppression as the main dose-limiting toxicity, and this may limit the future use of PARP inhibitors with chemotherapy (Chen, 2011).

Radiotherapy induces DNA damage by multiple mechanisms including base damage and single- and double-strand DNA breaks; damage that is dependent on PARP activity for its repair. Numerous *in vitro* and *in vivo* studies (Powell *et al*, 2010) using different classes of PARP inhibitors have reported enhancement of the cytotoxicity of radiation in a number of tumour types, including colorectal cancers (Calabrese *et al*, 2004) and gliomas (Dungey *et al*, 2009; Russo *et al*, 2009). More recently, work by Anthony Chalmers’ group has shown that this radio-potentiation is enhanced in rapidly proliferating cells and cells defective in DNA DSB repair compared with normal tissue (Loser *et al*, 2010). These data support a role for combining radiotherapy and PARP inhibitors in patients with cancer, and clinical trials are finally underway (www.clinicaltrials.gov) with results eagerly awaited.

## Summary

Poly (ADP-ribose) polymerase inhibitors are an exciting new development in the treatment of cancer, with clinical trials of single agents showing significant benefits in patients with *BRCA*m ovarian cancer. The mechanism underlying this benefit is the HRD of *BRCA*m cancers. Historically, germline *BRCA1/2* mutations were thought to be associated with approximately 10% of all ovarian cancers, but this is now known to be an underestimate. In addition, HRD is reported to be present in approximately 50% of all HGSOC cases. This suggests that the use of PARP inhibitors may have a much broader role in the treatment of ovarian cancer and the development of a validated HRD signature would facilitate this.

Finally, the recent licensing of olaparib in *BRCA*m ovarian cancer brings together over 50 years of research and is the first targeted treatment option for this patient population, taking another step further towards personalised medicine in ovarian cancer.

## Figures and Tables

**Table 1:** PARP inhibitors in Phase III clinical trial development for ovarian cancer, 2015

**Agent**	**Company**	**IC**_**50**_	**Ongoing clinical trials**	**Patient population**	**Indication**
Olaparib (AZD2281)	AstraZeneca	5 nM (PARP1) 1 nM (PARP2) (Menear *et al*, 2008)	SOLO1 (NCT01844986)	*BRCA*-mutated, advanced (FIGO Stage III–IV), high-grade serous/endometrioid; response (CR or PR) to initial platinum-based chemotherapy	First line
			SOLO2 (NCT01874353)	*BRCA*-mutated, high-grade serous/endometrioid; response (CR or PR) following ⩾2 lines of platinum-based chemotherapy	Relapsed
			SOLO3 (NCT02282020)	Germline *BRCA*-mutated, platinum-sensitive relapsed, high-grade serous/endometrioid	Relapsed
			SOLOiST (NCT02392676)	Platinum-sensitive relapsed, high-grade epithelial; deficient DNA damage repair (must not be caused by a germline *BRCA* mutation)	Relapsed
Niraparib (MK4827)	Merck (licensed to Tesaro)	3.8 nM (PARP1) 2.1 nM (PARP2) (Jones *et al*, 2009)	NOVA (NCT01847274)	*BRCA*-mutated or high-grade serous; sensitive to penultimate platinum-based regimen; response (CR or PR) to current platinum-based chemotherapy	Relapsed
Rucaparib (AG014699)	Clovis Oncology	1.4 nM (Ki; PARP1) 0.5 nM (Ki; PARP2) (Thomas *et al*, 2007)	ARIEL3 (NCT01968213)	High-grade serous/endometrioid; sensitive to penultimate platinum-based regimen; response (CR or PR) to current platinum-based chemotherapy	Relapsed
Talazoparib (BMN-673)	Medivation	0.58 nM (PARP1) (Shen *et al*, 2013)	None	—	—
Veliparib (ABT-888)	AbbVie and BMS	5.2 nM (PARP1) 2.9 nM (PARP2) (Donawho *et al*, 2007)	NCT02470585	Advanced (FIGO Stage III or IV), high-grade serous	First line

Abbreviations: CR=complete response; FIGO=Féderation Internationale de Gynécologie et d'Obstétrique; IC_50_=the concentration of a drug required for 50% inhibition; PR=partial response.

## References

[bib1] Alsop K, Fereday S, Meldrum C, deFazio A, Emmanuel C, George J, Dobrovic A, Birrer MJ, Webb PM, Stewart C, Friedlander M, Fox S, Bowtell D, Mitchell G (2012) BRCA mutation frequency and patterns of treatment response in BRCA mutation-positive women with ovarian cancer: a report from the Australian Ovarian Cancer Study Group. J Clin Oncol 30: 2654–2663. 22711857 10.1200/JCO.2011.39.8545 PMC3413277

[bib2] Audeh MW (2014) Novel treatment strategies in triple-negative breast cancer: specific role of poly(adenosine diphosphate-ribose) polymerase inhibition. Pharmgenomics Pers Med 7: 307–316. 25342917 10.2147/PGPM.S39765 PMC4205934

[bib3] Audeh MW, Carmichael J, Penson RT, Friedlander M, Powell B, Bell-McGuinn KM, Scott C, Weitzel JN, Oaknin A, Loman N, Lu K, Schmutzler RK, Matulonis U, Wickens M, Tutt A (2010) Oral poly(ADP-ribose) polymerase inhibitor olaparib in patients with *BRCA1* or *BRCA2* mutations and recurrent ovarian cancer: a proof-of-concept trial. Lancet 376: 245–251. 20609468 10.1016/S0140-6736(10)60893-8

[bib4] Benjamin RC, Gill DM (1980) ADP-ribosylation in mammalian cell ghosts. Dependence of poly(ADP-ribose) synthesis on strand breakage in DNA. J Biol Chem 255: 10493–10501. 7430132

[bib5] Bhattacharyya A, Ear US, Koller BH, Weichselbaum RR, Bishop DK (2000) The breast cancer susceptibility gene BRCA1 is required for subnuclear assembly of Rad51 and survival following treatment with the DNA cross-linking agent cisplatin. J Biol Chem 275: 23899–23903. 10843985 10.1074/jbc.C000276200

[bib6] Bolton KL, Chenevix-Trench G, Goh C, Sadetzki S, Ramus SJ, Karlan BY, Lambrechts D, Despierre E, Barrowdale D, McGuffog L, Healey S, Easton DF, Sinilnikova O, Benítez J, García MJ, Neuhausen S, Gail MH, Hartge P, Peock S, Frost D, Evans DG, Eeles R, Godwin AK, Daly MB, Kwong A, Ma ES, Lázaro C, Blanco I, Montagna M, D'Andrea E, Nicoletto MO, Johnatty SE, Kjær SK, Jensen A, Høgdall E, Goode EL, Fridley BL, Loud JT, Greene MH, Mai PL, Chetrit A, Lubin F, Hirsh-Yechezkel G, Glendon G, Andrulis IL, Toland AE, Senter L, Gore ME, Gourley C, Michie CO, Song H, Tyrer J, Whittemore AS, McGuire V, Sieh W, Kristoffersson U, Olsson H, Borg Å, Levine DA, Steele L, Beattie MS, Chan S, Nussbaum RL, Moysich KB, Gross J, Cass I, Walsh C, Li AJ, Leuchter R, Gordon O, Garcia-Closas M, Gayther SA, Chanock SJ, Antoniou AC, Pharoah PD EMBRACE; kConFab Investigators; Cancer Genome Atlas Research Network (2012) Association between BRCA1 and BRCA2 mutations and survival in women with invasive epithelial ovarian cancer. JAMA 307: 382–390. 22274685 10.1001/jama.2012.20 PMC3727895

[bib7] Bryant HE, Petermann E, Schultz N, Jemth AS, Loseva O, Issaeva N, Johansson F, Fernandez S, McGlynn P, Helleday T (2009) PARP is activated at stalled forks to mediate Mre11-dependent replication restart and recombination. EMBO J 28: 2601–2615. 19629035 10.1038/emboj.2009.206 PMC2738702

[bib8] Bryant HE, Schultz N, Thomas HD, Parker KM, Flower D, Lopez E, Kyle S, Meuth M, Curtin NJ, Helleday T (2005) Specific killing of BRCA2-deficient tumours with inhibitors of poly(ADP-ribose) polymerase. Nature 434: 913–917. 15829966 10.1038/nature03443

[bib9] Buisson R, Dion-Côté A-M, Coulombe Y, Launay H, Cai H, Stasiak AZ, Stasiak A, Xia B, Masson J-Y (2010) Cooperation of breast cancer proteins PALB2 and piccolo BRCA2 in stimulating homologous recombination. Nat Struct Mol Biol 17(10): 1247–1254. 20871615 10.1038/nsmb.1915 PMC4094107

[bib10] Calabrese CR, Almassy R, Barton S, Batey MA, Calvert AH, Canan-Koch S, Durkacz BW, Hostomsky Z, Kumpf RA, Kyle S, Li J, Maegley K, Newell DR, Notarianni E, Stratford IJ, Skalitzky D, Thomas HD, Wang LZ, Webber SE, Williams KJ, Curtin NJ (2004) Anticancer chemosensitization and radiosensitization by the novel poly(ADP-ribose) polymerase-1 inhibitor AG14361. J Natl Cancer Inst 96: 56–67. 14709739 10.1093/jnci/djh005

[bib11] Cancer Genome Atlas Research Network (2011) Integrated genomic analyses of ovarian carcinoma. Nature 474: 609–615. 21720365 10.1038/nature10166 PMC3163504

[bib12] Chambon P, Weill JD, Mandel P (1963) Nicotinamide mononucleotide activation of new DNA-dependent polyadenylic acid synthesizing nuclear enzyme. Biochem Biophys Res Commun 11: 39–43. 14019961 10.1016/0006-291x(63)90024-x

[bib13] Chen A (2011) PARP inhibitors: its role in treatment of cancer. Chin J Cancer 30: 463–471. 21718592 10.5732/cjc.011.10111 PMC4013421

[bib14] Chen S, Parmigiani G (2007) Meta-analysis of BRCA1 and BRCA2 penetrance. J Clin Oncol 25: 1329–1333. 17416853 10.1200/JCO.2006.09.1066 PMC2267287

[bib15] Delaney CA, Wang LZ, Kyle S, Kyle S, White AW, Calvert AH, Curtin NJ, Durkacz BW, Hostomsky Z, Newell DR (2000) Potentiation of temozolomide and topotecan growth inhibition and cytotoxicity by novel poly(adenosine diphosphoribose) polymerase inhibitors in a panel of human tumor cell lines. Clin Cancer Res 6: 2860–2867. 10914735

[bib16] Domchek SM, Friebel TM, Neuhausen SL, Wagner T, Evans G, Isaacs C, Garber JE, Daly MB, Eeles R, Matloff E, Tomlinson GE, Van't Veer L, Lynch HT, Olopade OI, Weber BL, Rebbeck TR (2006) Mortality after bilateral salpingo-oophorectomy in BRCA1 and BRCA2 mutation carriers: a prospective cohort study. Lancet Oncol 7: 223–229. 16510331 10.1016/S1470-2045(06)70585-X

[bib17] Donawho CK, Luo Y, Penning TD, Bauch JL, Bouska JJ, Bontcheva-Diaz VD, Cox BF, DeWeese TL, Dillehay LE, Ferguson DC, Ghoreishi-Haack NS, Grimm DR, Guan R, Han EK, Holley-Shanks RR, Hristov B, Idler KB, Jarvis K, Johnson EF, Kleinberg LR, Klinghofer V, Lasko LM, Liu X, Marsh KC, McGonigal TP, Meulbroek JA, Olson AM, Palma JP, Rodriguez LE, Shi Y, Stavropoulos JA, Tsurutani AC, Zhu GD, Rosenberg SH, Giranda VL, Frost DJ (2007) ABT-888, an orally active poly(ADP-ribose) polymerase inhibitor that potentiates DNA-damaging agents in preclinical tumor models. Clin Cancer Res 13: 2728–2737. 17473206 10.1158/1078-0432.CCR-06-3039

[bib18] Dungey FA, Caldecott KW, Chalmers AJ (2009) Enhanced radiosensitization of human glioma cells by combining inhibition of poly(ADP-ribose) polymerase with inhibition of heat shock protein 90. Mol Cancer Ther 8: 2243–2254. 19671736 10.1158/1535-7163.MCT-09-0201 PMC2728724

[bib19] Durkacz BW, Omidiji O, Gray DA, Shall S (1980) (ADP-ribose)n participates in DNA excision repair. Nature 283: 593–596. 6243744 10.1038/283593a0

[bib20] Evers B, Drost R, Schut E, de Bruin M, van der Burg E, Derksen PW, Holstege H, Liu X, van Drunen E, Beverloo HB, Smith GC, Martin NM, Lau A, O'Connor MJ, Jonkers J (2008) Selective inhibition of BRCA2-deficient mammary tumor cell growth by AZD2281 and cisplatin. Clin Cancer Res 14: 3916–3925. 18559613 10.1158/1078-0432.CCR-07-4953

[bib21] Farmer H, McCabe N, Lord CJ, Tutt AN, Johnson DA, Richardson TB, Santarosa M, Dillon KJ, Hickson I, Knights C, Martin NM, Jackson SP, Smith GC, Ashworth A (2005) Targeting the DNA repair defect in BRCA mutant cells as a therapeutic strategy. Nature 434: 917–921. 15829967 10.1038/nature03445

[bib22] Ferraris DV (2010) Evolution of poly(ADP-ribose) polymerase-1 (PARP-1) inhibitors. From concept to clinic. J Med Chem 53: 4561–4584. 20364863 10.1021/jm100012m

[bib23] Fong PC, Boss DS, Yap TA, Tutt A, Wu P, Mergui-Roelvink M, Mortimer P, Swaisland H, Lau A, O'Connor MJ, Ashworth A, Carmichael J, Kaye SB, Schellens JH, de Bono JS (2009) Inhibition of poly(ADP-ribose) polymerase in tumors from BRCA mutation carriers. N Engl J Med 361: 123–134. 19553641 10.1056/NEJMoa0900212

[bib24] Gelmon KA, Tischkowitz M, Mackay H, Swenerton K, Robidoux A, Tonkin K, Hirte H, Huntsman D, Clemons M, Gilks B, Yerushalmi R, Macpherson E, Carmichael J, Oza A (2011) Olaparib in patients with recurrent high-grade serous or poorly differentiated ovarian carcinoma or triple-negative breast cancer: a phase 2, multicentre, open-label, non-randomised study. Lancet Oncol 12: 852–861. 21862407 10.1016/S1470-2045(11)70214-5

[bib25] Gudmundsdottir K, Ashworth A (2006) The roles of BRCA1 and BRCA2 and associated proteins in the maintenance of genomic stability. Oncogene 25: 5864–5874. 16998501 10.1038/sj.onc.1209874

[bib26] Hall JM, Lee MK, Newman B, Morrow JE, Anderson LA, Huey B, King MC (1990) Linkage of early-onset familial breast cancer to chromosome 17q21. Science 250: 1684–1689. 2270482 10.1126/science.2270482

[bib27] Hanker LC, Loibl S, Burchardi N, Pfisterer J, Meier W, Pujade-Lauraine E, Ray-Coquard I, Sehouli J, Harter P, du Bois A AGO and GINECO study group (2012) The impact of second to sixth line therapy on survival of relapsed ovarian cancer after primary taxane/platinum-based therapy. Ann Oncol 23: 2605–2612. 22910840 10.1093/annonc/mds203

[bib28] Helleday T, Lo J, van Gent DC, Engelward BP (2007) DNA double-strand break repair: from mechanistic understanding to cancer treatment. DNA Repair (Amst) 6: 923–935. 17363343 10.1016/j.dnarep.2007.02.006

[bib29] Helleday T, Petermann E, Lundin C, Hodgson B, Sharma RA (2008) DNA repair pathways as targets for cancer therapy. Nat Rev Cancer 8: 193–204. 18256616 10.1038/nrc2342

[bib30] Hoeijmakers JH (2001) Genome maintenance mechanisms for preventing cancer. Nature 411: 366–374. 11357144 10.1038/35077232

[bib31] Hoeijmakers JH (2009) DNA damage, aging, and cancer. N Engl J Med 361: 1475–1485. 19812404 10.1056/NEJMra0804615

[bib32] Jacob DA, Bahra M, Langrehr JM, Boas-Knoop S, Stefaniak R, Davis J, Schumacher G, Lippert S, Neumann UP (2007) Combination therapy of poly (ADP-ribose) polymerase inhibitor 3-aminobenzamide and gemcitabine shows strong antitumor activity in pancreatic cancer cells. J Gastroenterol Hepatol 22: 738–748. 17444865 10.1111/j.1440-1746.2006.04496.x

[bib33] Jones P, Altamura S, Boueres J, Ferrigno F, Fonsi M, Giomini C, Lamartina S, Monteagudo E, Ontoria JM, Orsale MV, Palumbi MC, Pesci S, Roscilli G, Scarpelli R, Schultz-Fademrecht C, Toniatti C, Rowley M (2009) Discovery of 2-{4-[(3S)-piperidin-3-yl]phenyl}-2H-indazole-7-carboxamide (MK-4827): a novel oral poly(ADP-ribose)polymerase (PARP) inhibitor efficacious in BRCA-1 and -2 mutant tumors. J Med Chem 52: 7170–7185. 19873981 10.1021/jm901188v

[bib34] Kaelin WG Jr (2005) The concept of synthetic lethality in the context of anticancer therapy. Nat Rev Cancer 5: 689–698. 16110319 10.1038/nrc1691

[bib35] Kaufman B, Shapira-Frommer R, Schmutzler RK, Audeh MW, Friedlander M, Balmaña J, Mitchell G, Fried G, Stemmer SM, Hubert A, Rosengarten O, Steiner M, Loman N, Bowen K, Fielding A, Domchek SM (2015) Olaparib monotherapy in patients with advanced cancer and a germ-line *BRCA1/2* mutation. J Clin Oncol 33: 244–250. 25366685 10.1200/JCO.2014.56.2728 PMC6057749

[bib36] Leavy O (2010) V(D)J recombination: RAG recombination centres. Nat Rev Immunol 10: 383. 20514673 10.1038/nri2789

[bib37] Ledermann JA, El-Khouly F (2015) PARP inhibitors in ovarian cancer: clinical evidence for informed treatment decisions. Br J Cancer S1–S7 doi:10.1038/bjc.2015.395. 10.1038/bjc.2015.395 PMC4816268 26669450

[bib38] Ledermann JA, Harter P, Gourley C, Friedlander M, Vergote I, Rustin G, Scott CL, Meier W, Shapira-Frommer R, Safra T, Matei D, Fielding A, Spencer S, Dougherty B, Orr M, Hodgson D, Barrett JC, Matulonis U (2014) Olaparib maintenance therapy in patients with platinum-sensitive relapsed serous ovarian cancer: a preplanned retrospective analysis of outcomes by BRCA status in a randomised phase 2 trial. Lancet Oncol 15: 852–861. 24882434 10.1016/S1470-2045(14)70228-1

[bib39] Liu JF, Barry WT, Birrer M, Lee JM, Buckanovich RJ, Fleming GF, Rimel B, Buss MK, Nattam S, Hurteau J, Luo W, Quy P, Whalen C, Obermayer L, Lee H, Winer EP, Kohn EC, Ivy SP, Matulonis UA (2014) Combination cediranib and olaparib versus olaparib alone for women with recurrent platinum-sensitive ovarian cancer: a randomised phase 2 study. Lancet Oncol 15: 1207–1214. 25218906 10.1016/S1470-2045(14)70391-2 PMC4294183

[bib40] Loser DA, Shibata A, Shibata AK, Woodbine LJ, Jeggo PA, Chalmers AJ (2010) Sensitization to radiation and alkylating agents by inhibitors of poly(ADP-ribose) polymerase is enhanced in cells deficient in DNA double-strand break repair. Mol Cancer Ther 9: 1775–1787. 20530711 10.1158/1535-7163.MCT-09-1027 PMC2884153

[bib41] McNeish I, Coleman RL, Oza A, Konecny GE, O'Malley D, Kichendasse G, Scott C, Oaknin A, Floquet A, Park D, Brenton J, Lin K, Shetty S, Raponi M, Isaacson J, Rolfe L, Giordano H, Allen A, Swisher E (2014) Preliminary results of ARIEL2, a phase 2 open-label study to identify ovarian cancer patients likely to respond to rucaparib. European Society for Medical Oncology (ESMO) Annual Meeting, Madrid, Spain ; 26–30 September; abstract 883PD.

[bib42] Menear KA, Adcock C, Boulter R, Cockcroft XL, Copsey L, Cranston A, Dillon KJ, Drzewiecki J, Garman S, Gomez S, Javaid H, Kerrigan F, Knights C, Lau A, Loh VM Jr, Matthews IT, Moore S, O'Connor MJ, Smith GC, Martin NM (2008) 4-[3-(4-Cyclopropanecarbonylpiperazine-1-carbonyl)-4-fluorobenzyl]-2H-phth alazin-1-one: a novel bioavailable inhibitor of poly(ADP-ribose) polymerase-1. J Med Chem 51: 6581–6591. 18800822 10.1021/jm8001263

[bib43] Miki Y, Swensen J, Shattuck-Eidens D, Futreal PA, Harshman K, Tavtigian S, Liu Q, Cochran C, Bennett M, Ding W, Bell R, Rosenthal J, Hussey C, Tran T, McClure M, Frye C, Hattier T, Phelps R, Haugen-Strano A, Katcher H, Yakumo K, Gholami Z, Schaffer D, Stone S, Bayer S, Wray C, Bogden R, Dayananth P, Ward J, Tonin P, Narod S, Bristow PK, Norris FH, Helvering L, Morrison P, Rosteck P, Lai M, Barrett JC, Lewis C, Neuhausen S, Cannon-Albright L, Goldgar D, Wiseman R, Kamb A, Skolnick MH (1994) A strong candidate for the breast and ovarian cancer susceptibility gene BRCA1. Science 266: 66–71. 7545954 10.1126/science.7545954

[bib44] Mukhopadhyay A, Elattar A, Cerbinskaite A, Wilkinson SJ, Drew Y, Kyle S, Los G, Hostomsky Z, Edmondson RJ, Curtin NJ (2010) Development of a functional assay for homologous recombination status in primary cultures of epithelial ovarian tumor and correlation with sensitivity to poly(ADP-ribose) polymerase inhibitors. Clin Cancer Res 16: 2344–2351. 20371688 10.1158/1078-0432.CCR-09-2758

[bib45] Neale MJ, Keeney S (2006) Clarifying the mechanics of DNA strand exchange in meiotic recombination. Nature 442: 153–158. 16838012 10.1038/nature04885 PMC5607947

[bib46] O'Driscoll M, Jeggo PA (2006) The role of double-strand break repair—insights from human genetics. Nat Rev Genet 7: 45–54. 16369571 10.1038/nrg1746

[bib47] Oza AM, Cibula D, Benzaquen AO, Poole C, Mathijssen RH, Sonke GS, Colombo N, Špaček J, Vuylsteke P, Hirte H, Mahner S, Plante M, Schmalfeldt B, Mackay H, Rowbottom J, Lowe ES, Dougherty B, Barrett JC, Friedlander M (2015) Olaparib combined with chemotherapy for recurrent platinum-sensitive ovarian cancer: a randomised phase 2 trial. Lancet Oncol 16: 87–97. 25481791 10.1016/S1470-2045(14)71135-0

[bib48] Powell C, Mikropoulos C, Kaye SB, Nutting CM, Bhide SA, Newbold K, Harrington KJ (2010) Pre-clinical and clinical evaluation of PARP inhibitors as tumour-specific radiosensitisers. Cancer Treat Rev 36: 566–575. 20409643 10.1016/j.ctrv.2010.03.003

[bib49] Purnell MR, Whish WJ (1980) Novel inhibitors of poly(ADP-ribose) synthetase. Biochem J 185: 775–777. 6248035 10.1042/bj1850775 PMC1161458

[bib50] Risch HA, McLaughlin JR, Cole DE, Rosen B, Bradley L, Fan I, Tang J, Li S, Zhang S, Shaw PA, Narod SA (2006) Population BRCA1 and BRCA2 mutation frequencies and cancer penetrances: a kin-cohort study in Ontario, Canada. J Natl Cancer Inst 98: 1694–1706. 17148771 10.1093/jnci/djj465

[bib51] Risch HA, McLaughlin JR, Cole DE, Rosen B, Bradley L, Kwan E, Jack E, Vesprini DJ, Kuperstein G, Abrahamson JL, Fan I, Wong B, Narod SA (2001) Prevalence and penetrance of germline BRCA1 and BRCA2 mutations in a population series of 649 women with ovarian cancer. Am J Hum Genet 68: 700–710. 11179017 10.1086/318787 PMC1274482

[bib52] Rottenberg S, Jaspers JE, Kersbergen A, van der Burg E, Nygren AO, Zander SA, Derksen PW, de Bruin M, Zevenhoven J, Lau A, Boulter R, Cranston A, O'Connor MJ, Martin NM, Borst P, Jonkers J (2008) High sensitivity of BRCA1-deficient mammary tumors to the PARP inhibitor AZD2281 alone and in combination with platinum drugs. Proc Natl Acad Sci USA 105: 17079–17084. 18971340 10.1073/pnas.0806092105 PMC2579381

[bib53] Rouleau M, Patel A, Hendzel MJ, Kaufmann SH, Poirier GG (2010) PARP inhibition: PARP1 and beyond. Nat Rev Cancer 10: 293–301. 20200537 10.1038/nrc2812 PMC2910902

[bib54] Russo AL, Kwon HC, Burgan WE, Carter D, Beam K, Weizheng X, Zhang J, Slusher BS, Chakravarti A, Tofilon PJ, Camphausen K (2009) In vitro and in vivo radiosensitization of glioblastoma cells by the poly (ADP-ribose) polymerase inhibitor E7016. Clin Cancer Res 15: 607–612. 19147766 10.1158/1078-0432.CCR-08-2079 PMC6322204

[bib55] Saito Y, Fujimoto H, Kobayashi J (2013) Role of NBS1 in DNA damage response and its relationship with cancer development. Transl Cancer Res 2013 2(3): 178–189.

[bib56] Scott CL, Swisher EM, Kaufmann SH (2015) Poly (ADP-ribose) polymerase inhibitors: recent advances and future development. J Clin Oncol 33: 1397–1406. 25779564 10.1200/JCO.2014.58.8848 PMC4517072

[bib57] Shen Y, Rehman FL, Feng Y, Boshuizen J, Bajrami I, Elliott R, Wang B, Lord CJ, Post LE, Ashworth A (2013) BMN 673, a novel and highly potent PARP1/2 inhibitor for the treatment of human cancers with DNA repair deficiency. Clin Cancer Res 19: 5003–5015. 23881923 10.1158/1078-0432.CCR-13-1391 PMC6485449

[bib58] Siegel R, Naishadham D, Jemal A (2012) Cancer statistics, 2012. CA Cancer J Clin 62: 10–29. 22237781 10.3322/caac.20138

[bib59] Takata M, Sasaki MS, Sonoda E, Morrison C, Hashimoto M, Utsumi H, Yamaguchi-Iwai Y, Shinohara A, Takeda S (1998) Homologous recombination and non-homologous end-joining pathways of DNA double-strand break repair have overlapping roles in the maintenance of chromosomal integrity in vertebrate cells. EMBO J 17: 5497–5508. 9736627 10.1093/emboj/17.18.5497 PMC1170875

[bib60] Tan DS, Rothermundt C, Thomas K, Bancroft E, Eeles R, Shanley S, Ardern-Jones A, Norman A, Kaye SB, Gore ME (2008) ‘BRCAness’ syndrome in ovarian cancer: a case-control study describing the clinical features and outcome of patients with epithelial ovarian cancer associated with BRCA1 and BRCA2 mutations. J Clin Oncol 26: 5530–5536. 18955455 10.1200/JCO.2008.16.1703

[bib61] Tassone P, Tagliaferri P, Perricelli A, Blotta S, Quaresima B, Martelli ML, Goel A, Barbieri V, Costanzo F, Boland CR, Venuta S (2003) BRCA1 expression modulates chemosensitivity of BRCA1-defective HCC1937 human breast cancer cells. Br J Cancer 88: 1285–1291. 12698198 10.1038/sj.bjc.6600859 PMC2747554

[bib62] Thomas HD, Calabrese CR, Batey MA, Canan S, Hostomsky Z, Kyle S, Maegley KA, Newell DR, Skalitzky D, Wang LZ, Webber SE, Curtin NJ (2007) Preclinical selection of a novel poly(ADP-ribose) polymerase inhibitor for clinical trial. Mol Cancer Ther 6: 945–956. 17363489 10.1158/1535-7163.MCT-06-0552

[bib63] Tutt A, Robson M, Garber JE, Domchek SM, Audeh MW, Weitzel JN, Friedlander M, Arun B, Loman N, Schmutzler RK, Wardley A, Mitchell G, Earl H, Wickens M, Carmichael J (2010) Oral poly(ADP-ribose) polymerase inhibitor olaparib in patients with *BRCA1* or *BRCA2* mutations and advanced breast cancer: a proof-of-concept trial. Lancet 376: 235–244. 20609467 10.1016/S0140-6736(10)60892-6

[bib64] Venkitaraman AR (2002) Cancer susceptibility and the functions of BRCA1 and BRCA2. Cell 108: 171–182. 11832208 10.1016/s0092-8674(02)00615-3

[bib65] Virag L, Szabo C (2002) The therapeutic potential of poly(ADP-ribose) polymerase inhibitors. Pharmacol Rev 54: 375–429. 12223530 10.1124/pr.54.3.375

[bib66] Wooster R, Neuhausen SL, Mangion J, Quirk Y, Ford D, Collins N, Nguyen K, Seal S, Tran T, Averill D et al (1994) Localization of a breast cancer susceptibility gene, BRCA2, to chromosome 13q12-13. Science 265: 2088–2090. 8091231 10.1126/science.8091231

[bib67] World Health Organization (2008) GLOBOCAN statistics. Available at http://globocan.iarc.fr/.

[bib68] Yang H, Jeffrey PD, Miller J, Kinnucan E, Sun Y, Thoma NH, Zheng N, Chen PL, Lee WH, Pavletich NP (2002) BRCA2 function in DNA binding and recombination from a BRCA2-DSS1-ssDNA structure. Science 297: 1837–1848. 12228710 10.1126/science.297.5588.1837

[bib69] Zhong Q, Peng HL, Zhao X, Zhang L, Hwang WT (2015) Effects of BRCA1- and BRCA2-related mutations on ovarian and breast cancer survival: a meta-analysis. Clin Cancer Res 21: 211–220. 25348513 10.1158/1078-0432.CCR-14-1816 PMC4286460

